# High Surface Area Nanoporous Graphitic Carbon Materials Derived from Lapsi Seed with Enhanced Supercapacitance

**DOI:** 10.3390/nano10040728

**Published:** 2020-04-11

**Authors:** Lok Kumar Shrestha, Rekha Goswami Shrestha, Subrata Maji, Bhadra P. Pokharel, Rinita Rajbhandari, Ram Lal Shrestha, Raja Ram Pradhananga, Jonathan P. Hill, Katsuhiko Ariga

**Affiliations:** 1 International Center for Materials Nanoarchitectonics (WPI-MANA), National Institute for Materials Science (NIMS), 1-1 Namiki, Tsukuba 305-0044, Japan; MAJI.Subrata@nims.go.jp (S.M.); Jonathan.HILL@nims.go.jp (J.P.H.); ariga.katsuhiko@nims.go.jp (K.A.); 2 Materials Science and Engineering Program, Pulchowk Campus, Institute of Engineering (IOE), Tribhuvan University (TU), Lalitpur, Kathmandu 44700, Nepal; bhadrapokharel@ioe.edu.np (B.P.P.); joshirinita@yahoo.com (R.R.); 3 Amrit Campus, Tribhuvan University, Kathmandu 44613, Nepal; swagatstha@gmail.com (R.L.S.); rajaram2620@gmail.com (R.R.P.); 4 Graduate School of Frontier Sciences, the University of Tokyo, 5-1-5 Kashiwanoha, Kashiwa, Chiba 277-8561, Japan

**Keywords:** Lapsi seed, chemical activation, microporous activated carbon, supercapacitor, high rate capability, excellent cyclic stability

## Abstract

Nanoporous activated carbon materials derived from agro-wastes could be suitable low-cost electrode materials for high-rate performance electrochemical supercapacitors. Here we report high surface area nanoporous carbon materials derived from Lapsi seed agro-waste prepared by zinc chloride (ZnCl_2_) activation at 700 °C. Powder X-ray diffraction (pXRD) and Raman scattering confirmed the amorphous structure of the resulting carboniferous materials, which also incorporate oxygen-containing functional groups as confirmed by Fourier transform infrared (FTIR) spectroscopy. Scanning and transmission electron microscopy (SEM and TEM) analyses revealed the granular, nanoporous structures of the materials. High-resolution TEM (HR-TEM) confirmed a graphitic carbon structure containing interconnected mesopores. Surface areas and pore volumes of the materials were found, respectively, in the ranges from 931 to 2272 m^2^ g^−1^ and 0.998 to 2.845 cm^3^ g^−1^, and are thus superior to commercially available activated carbons. High surface areas, large pore volumes and interconnected mesopore structures of these Lapsi seed-derived nanoporous carbon materials lead to their excellent electrochemical supercapacitance performance in aqueous electrolyte (1 M H_2_SO_4_) with a maximum specific capacitance of 284 F g^−1^ at a current density of 1 A g^−1^. Furthermore, the electrodes showed high-rate capability sustaining 67.7% capacity retention even at high current density of 20 A g^−1^ with excellent cycle stability achieving 99% capacitance retention even after 10,000 charge–discharge cycles demonstrating the potential of Lapsi seed derived nanoporous carbons as suitable electrode materials in high-performance supercapacitor devices.

## 1. Introduction

Ultracapacitors or electrical double-layer capacitors (EDLCs) are of substantial technological interest since they provide improvements in operation over the poor cycle stabilities and low power densities of conventional rechargeable batteries [1,2,3,4,5]. Supercapacitors are currently in use as electrochemical energy storage systems in portable electronic devices and memory backup systems [6,7,8,9,10]. Supercapacitors are also used as auxiliary power sources for the starting and acceleration of high-performance automobiles or for the operation of electric buses [11,12]. However, a major drawback of supercapacitors is their relatively low energy density (1–10 Wh kg^−1^) compared to lead-acid (30–40 Wh kg^−1^) or lithium ion batteries (160 Wh kg^−1^) [13,14]. Therefore, extensive research has been carried out to enhance the energy density of supercapacitors. It can be improved either by increasing the specific capacitance of the active electrode material or by widening the operating voltage window [15]. The operating potential window can be increased by replacing the aqueous electrolyte with a non-aqueous electrolyte such as an ionic liquid [16,17,18]. Nevertheless, for practical uses of supercapacitor devices, an aqueous electrolyte is usually preferred for economic and safety reasons. Therefore, more attention is paid to improve the specific capacitances of the materials. Previous investigations have shown that specific capacitance of an electrode material is correlated directly with its surface textural properties including specific surface area, pore volume, interconnectedness of pores and the conductivity of the materials indicating the importance of the design of the electrode materials [19,20]. Other factors affecting their preparation are relative expense and environmental friendliness of the electrode materials [21,22,23,24].

Nanoporous carbon materials represent the best electrode materials for commercial supercapacitors due to their excellent cycle stability, good rate capability, wide range of operating voltage, and cost-effective synthesis protocols. Various nanocarbon materials such as graphene, carbon nanotubes (CNTs) [25,26], fullerenes [27,28] and various other forms, such as activated carbons [29,30], template assisted mesoporous carbons [31], carbide-derived carbons [32,33] have been explored as electrode materials for supercapacitor applications. However, nanoscale carbons have their own limitations including inherent low conductivities of fullerene-based materials [34] or reduced in specific surface areas for graphene-based materials caused by π-π stacking [35,36]. Several strategies have been adopted to overcome these limitations including high temperature heat treatment for fullerenes [37,38], or surface functionalization for graphenes [39]. Extra processing steps lead to an increase in production costs, which limits their industrial-scale applications as electrode materials for supercapacitors. Template-assisted synthesis of mesoporous carbons is of interest since it allows the preparation of materials of high surface area and tunable porosity. However, large scale production is less feasible because of inconvenient multistep syntheses and the use of a sacrificial template. On the other hand, nanoporous-activated carbon materials have been extensively used as EDLC electrode materials because of their micropore-enabled high specific surface areas, large pore volumes and, most importantly, simple and cost-effective fabrication processes [40,41,42]. Extensive efforts have been made in the cost-effective production of high surface area nanoporous carbon materials by using various agricultural wastes or biomass as precursor materials. Biomass precursors such as pitch [43], pistachio shell [44], rice husks [45,46], coconut shell [47], eucalyptus wood [48], firewood [49,50], oil-palm shell [51,52], babassu [53], corncob [54], bamboo [53], corn husks [54] etc. have been investigated as carbonaceous precursors using two major synthesis protocols—direct carbonization followed by activation and carbonization/activation in a single step. In particular, the interconnected mesoporous network structures with open pores of biomass-derived nanocarbon materials with three-dimensional (3D) architecture obtained by an activation process offer easy access for the guest ions to their interiors thus increasing the charge storage capacity of the materials by increasing the effective surface area accessible to the electrolyte ions. While activating agents such sulfuric acid, phosphoric acid, potassium hydroxide, or potassium carbonate have been used, zinc chloride (ZnCl_2_) is one of the most extensively used agents for the chemical activation of a carbonaceous material [55,56,57]. ZnCl_2_ acts as a dehydrating agent and accelerates the decomposition of carbonaceous materials during the carbonization process and restricts the formation of tar. Therefore, the yields of nanoporous carbon materials obtained by ZnCl_2_ activation are always high compared to those obtained with other activating agents. Previously, we have fabricated nanoporous carbon material by the ZnCl_2_ activation of Lapsi seed powder at a relatively lower temperature, 400 °C. The carbon materials achieved surface areas in the range of 1167 to 1328 m^2^ g^−1^ [58] and the materials showed excellent arsenic removal from groundwater. Similarly, we had also prepared nanoporous carbon from Lapsi seed by sodium hydroxide activation and studied the effect of carbonization conditions such as impregnation ratio of sodium hydroxide, carbonization temperature, and carbonization time [59]. In sodium hydroxide activation, the optimal sample displayed a specific surface area about 1000 m^2^ g^−1^ and the material showed excellent dye adsorption properties.

In this contribution, we report the facile fabrication of very high surface area nanoporous activated carbon materials from Lapsi seed agro-waste by the ZnCl_2_ activation method, and their use as electrode materials for high-rate performance supercapacitors in aqueous electrolyte. Our synthetic method includes chemical activation of Lapsi seed powder at a moderate carbonization temperature (700 °C). Lapsi seed-derived carbons exhibit hierarchical micro- and mesoporous structures with the optimal sample displaying a very high surface area of 2272 m^2^ g^−1^. Because of the high surface areas, large pore volumes, and interconnected mesopore structure, Lapsi carbon exhibited excellent electrochemical supercapacitance performance in aqueous electrolyte (1 M H_2_SO_4_) giving maximum specific capacitance of 284 F g^−1^ at current density 1 A g^−1^ accompanied by high-rate capability sustaining 67.7% capacity retention even at high current density of 20 A g^−1^ and excellent cycle stability achieving 99% capacitance retention even after 10,000 charge–discharge cycles. This work demonstrates utilization of biomass for the production of excellent electrode materials for high-performance supercapacitor devices.

## 2. Experimental

### 2.1. Preparation of Activated Carbons

Lapsi seed powder was chemically activated using ZnCl_2_ as activating agent. Lapsi seed powder was mixed with ZnCl_2_ at different weight ratios. Carbonization and activation was performed at 700 °C in a tubular furnace (KOYO, Tokyo, Japan) under a continuous flow of nitrogen (120 cc min^−1^) for 4 h. The carbonized samples were washed thoroughly with distilled water and the resulting activated nanoporous carbon materials were dried under reduced pressure at 80 °C for 6 h. The products were ground into fine powders prior to characterization and electrochemical studies. The weight ratios of Lapsi seed powder and ZnCl_2_ were 1:0, 1:0.5, 1:1, 1:2, 1:4 and the corresponding carbons obtained were referred to as **LSC_0**, **LSC_0.5**, **LSC_1**, **LSC_2** and **LSC_4**. 

### 2.2. Characterizations

Powder X-ray diffraction (XRD) patterns were recorded on a Rigaku X-ray diffractometer, RINT, Japan, operated at 40 kV and 40 mA with Cu-K_α_ radiation at 25 °C in the range 10 to 50°. Raman scattering spectra were recorded on a Jobin-Yvon T64000 Raman spectrometer with a green laser of wavelength 514.5 nm at 0.01 mW power. Raman samples were prepared by placing several carbon particles on a glass substrate. The presence of surface functional groups of the nanoporous carbons was confirmed by recording Fourier transform infrared (FTIR) spectra using a Nicolet 4700 (Thermo Electron Corporation, Waltham, MA, USA) at 25 °C. FTIR spectra were measured from KBr pellets of the samples in transmission mode from 400–4000 cm^−1^. Scanning electron microscopy (SEM) was used to study the surface morphology and porous structures of the samples. Samples for SEM were prepared on carbon tape and images recorded on an S-4800, Hitachi Co., Ltd. Japan at an operating voltage of 10 kV. SEM samples were platinum-coated (~2 nm) using a Hitachi S-2030 ion coater to avoid sample charging effects. For characterize the interconnectedness of nanoporosity, transmission electron microscopy (TEM) was used. TEM, high-resolution TEM (HR-TEM) images and selected area electron diffraction (SAED) patterns were recorded from the smallest components of the samples on a JEOL Model JEM2100F operating at 200 kV. Samples for TEM were prepared by dropping a dilute suspension of the carbon sample in isopropanol onto a carbon-coated copper grid followed by drying under reduced pressure for 24 h prior to TEM observations. Nitrogen adsorption/desorption isotherms were recorded on an automatic adsorption instrument (Quantachrome Autosorb-iQ2, Boynton Beach, FL, USA) and specific surface area, pore volume and pore size distribution were determined. For each measurement ~15 mg of sample was degassed for 24 h at 120 °C prior to measurement. Isotherms were recorded at liquid nitrogen temperature 77.35 K. 

### 2.3. Electrochemical Measurements

Electrochemical performances of Lapsi seed derived nanoporous carbon materials were measured by using cyclic voltammetry (CV) with a three-electrode system in 1 M aqueous H_2_SO_4_ solution at 25 °C. A modified bare glassy carbon electrode (GCE) was used as the working electrode. GCE was mirror polished with alumina (Al_2_O_3_) slurry and cleaned with double-distilled water then sonicated in acetone for 5 min. For the preparation of working electrode, carbon material was dispersed in a mixture of water and ethanol (4:1) (1 mg mL^−1^) followed by sonication for 30 min. The resulting suspension (5 µL) was dropcast onto the GCE surface followed by drying at 60 °C for 2 h. After the solvent had evaporated, Nafion solution (5 µL: 5% in ethanol) was added as binder followed by drying at 80 °C for 12 h under reduced pressure. Platinum wire was used as a counter electrode and Ag/AgCl as the reference electrode. The cyclic voltammetry response and chronopotentiometry were performed on a CH instruments (CHI 850D Work station) from USA. 

Specific capacitance of the electrode material was calculated from charge–discharge (chronopotentiometry) curves using the following equation:

(1) $$C_{S}=\frac{It}{m\times \Delta V}$$

where *I* is the discharge current (A), *t* is the discharge time (s), *m* is the mass of active electrode materials and ∆*V* is the potential window.

## 3. Results and Discussion

**Figure 1:**
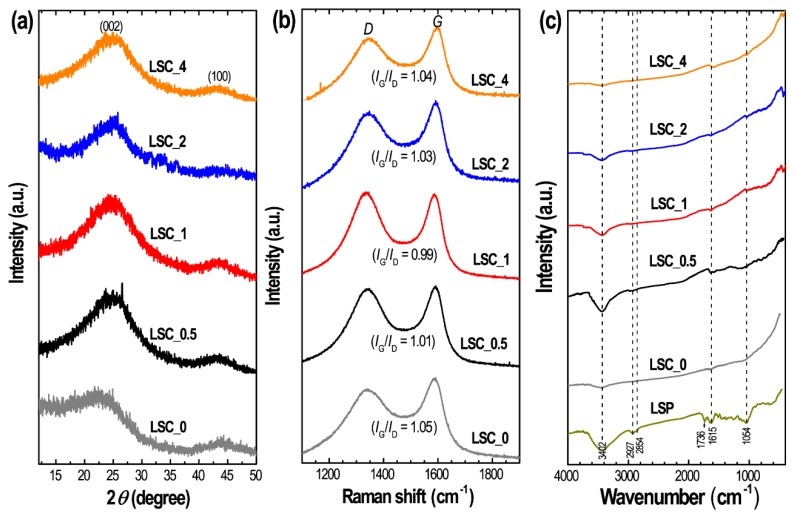
(**a**) Powder X-ray diffraction (XRD) patterns, (**b**) Raman scattering spectra and (**c**) Fourier transform infrared (FTIR) spectra of **LSC_0**, **LSC_0.5**, **LSC_1**, **LSC_2** and **LSC_4**. In panel (**c**), the FTIR spectrum of Lapsi seed powder (LSP) is also included.

Figure 1a shows powder X-ray diffraction patterns of Lapsi seed derived nanoporous carbon materials (**LSC_0**, **LSC_0.5**, **LSC_1**, **LSC_2** and **LSC_4**) recorded at 25 °C. All the samples show two broad diffraction peaks at angles 24° and 43° that correspond to the (002) and (100) planes of graphitic carbon characteristic of activated carbons prepared from other precursors. The relatively strong peak at 24° indicates the formation of amorphous carbons with random arrangement while the weak peak at 43° indicates the formation of a graphitic carbon structure [2,3,4].

Raman scattering spectra show two broad peaks around 1335 and 1595 cm^−1^, which are typical of amorphous carbon (Figure 1b). The peak at ~1335 cm^−1^ corresponds to the *D* band of the breathing mode vibration of A_1g_. The *G* band observed at ~1595 cm^−1^ corresponds to the in-plane stretching vibration mode of E_2g_ in *sp*^2^ carbons. For the estimation of the extent of graphitization, we have evaluated the intensity ratio (*I*_G_/*I*_D_) of *G* and *D* bands. The *I*_G_/*I*_D_ ratio of the prepared samples are found in the range of 0.99 to 1.05 typical of activated graphitic carbons [60]. The directly carbonized sample **LSC_0** displays the highest graphitization compared to the ZnCl_2_ activated carbons. For the confirmation of surface functional group present in the prepared carbon materials, FTIR spectra were recorded. It is well known that agro-waste precursors contain different hetero atom based functional groups. Here, Lapsi seed powder also contains oxygen-containing surface functional groups (Figure 1c). However, most of the functional groups are eliminated during the high-temperature activation process. FTIR spectra of the samples are similar containing a broad band in the range 3200–3600 cm^−1^ (centered at 3435 cm^−1^) which confirms the presence of O–H functional groups perhaps due to residual hydroxyl groups or adsorbed moisture [46]. A weak peak around 1634 cm^−1^ can be assigned to aromatic C=C stretching vibration [46].

**Figure 2:**
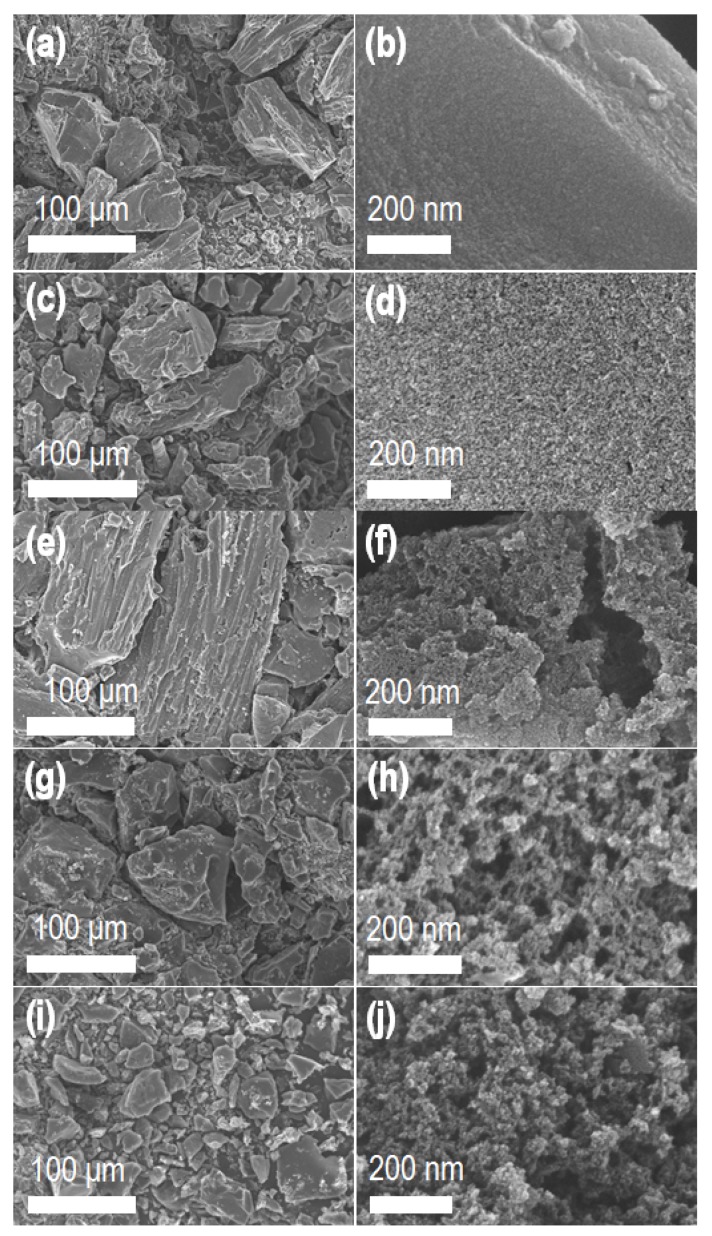
Scanning electron microscope (SEM) images of nanoporous carbon materials. (**a**,**b**) **LSC_0**, (**c**,**d**) **LSC_0.5**, (**e**,**f**) **LSC_1**, (**g**,**h**) **LSC_2** and (**i**,**j**) **LSC_4**.

Surface morphology and porous structures of the nanoporous carbons were studied by SEM. SEM images of carbon samples reveal the porous structure (Figure 2). Low-resolution SEM images (Figure 2a,c,e,g,i) show carbon granules of an irregular size distribution (50–300 μm) containing macropores. On the other hand, interconnected mesoporous architecture can be seen in the high-resolution SEM images of ZnCl_2_ activated samples (Figure 2d,f,h,j). We noted that directly carbonized sample, **LSC_0** does not have a significant number of mesopores (Figure 2b). Formation of a nanoporous structure can be attributed due to the release of moisture and volatile organic functional groups from the precursor material during the carbonization process.

**Figure 3:**
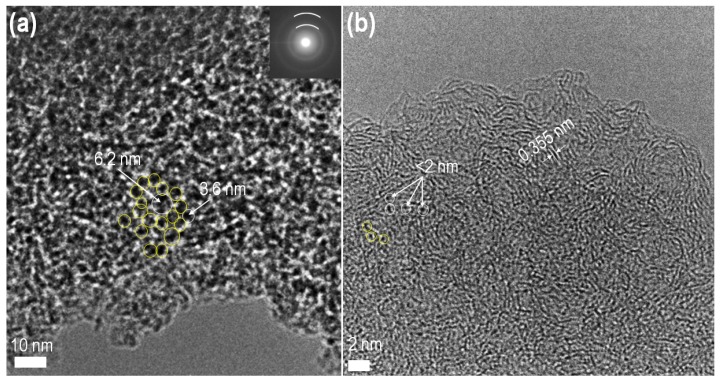
(**a**) Transmission electron microscope (TEM) image of **LSC_1** as a typical example. (**b**) The corresponding high-resolution TEM (HR-TEM) image. Inset of panel (**a**) shows the selected area electron diffraction (SAED) pattern. Circles in panels (**a**,**b**) highlight mesopores and micropores, respectively.

The well-developed and interconnected mesoporous structure can be better observed by TEM (Figure 3a). Mesopores as highlighted by circles in the TEM image. This mesoporous architecture should contribute to the rapid electrolyte ion diffusion, thus enhancing the rate performance of the electrode material. The selected area electron diffraction (SAED) pattern shown in the inset of Figure 3a displays two broad, weak circular rings (highlighted with white lines) indicating the amorphous nature of the Lapsi-derived carbon material [60]. The HR-TEM image shows microporous carbon (highlighted by circles) with the randomly grown graphitic carbon layer with interlayer spacing of 0.355 nm (Figure 3b).

**Figure 4:**
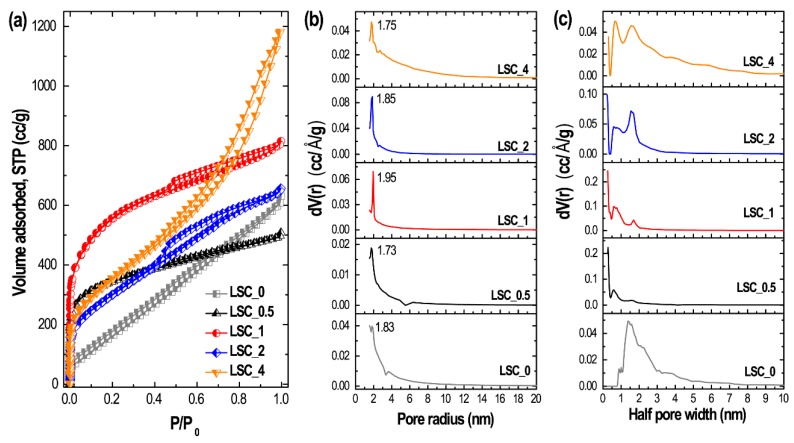
(**a**) Nitrogen adsorption/desorption isotherm of **LSC_0.5**, **LSC_1**, **LSC_2** and **LSC_4**, (**b**) pore size distribution obtained by the Barrett–Joyner–Halenda (BJH) method and (**c**) pore size distribution obtained by the density functional theory (DFT) method.

Surface textural properties (surface area, porosity and pore size distribution) of the nanoporous carbon materials prepared here were determined by recording nitrogen adsorption-desorption isotherms. Figure 4 shows the sorption isotherms and pore size distributions estimated by the Barrett–Joyner–Halenda (BJH) method and density functional theory (DFT). As can be seen in Figure 4a, the sorption isotherms exhibit mixed Type-I/Type IV behavior indicating the presence a microporous structure and also a hierarchical micro- and mesoporous architecture depending on the mixing ratio of the activating agent ZnCl_2_. 

In case of direct carbonization in the absence of activating agent, the isotherm of **LSC_0** indicates a disordered macroporous structure. **LSC_0.5** exhibits a Type-I adsorption isotherm where maximum nitrogen adsorption takes place at low relative pressure followed by almost complete saturation of nitrogen adsorption at higher relative pressure. High nitrogen adsorption at very low relative pressure indicates micropore (<2 nm) filling which confirms the microporosity of **LSC_0.5**. On the other hand, isotherms of **LSC_1**, **LSC_2** and **LSC_4** display significant quantities of nitrogen adsorption at lower relative pressure followed by a hysteresis loop at higher relative pressures (Figure 4a), which is a typical of porous materials possessing a hierarchical micro- and mesopore structure. Pore size distributions obtained by the BJH (Figure 4b) and DFT methods (Figure 4c) also confirm the presence of micropores and mesopores. The porosity properties of the prepared carbon materials obtained from the nitrogen sorption measurements are summarized in Table 1. 

**Figure 5:**
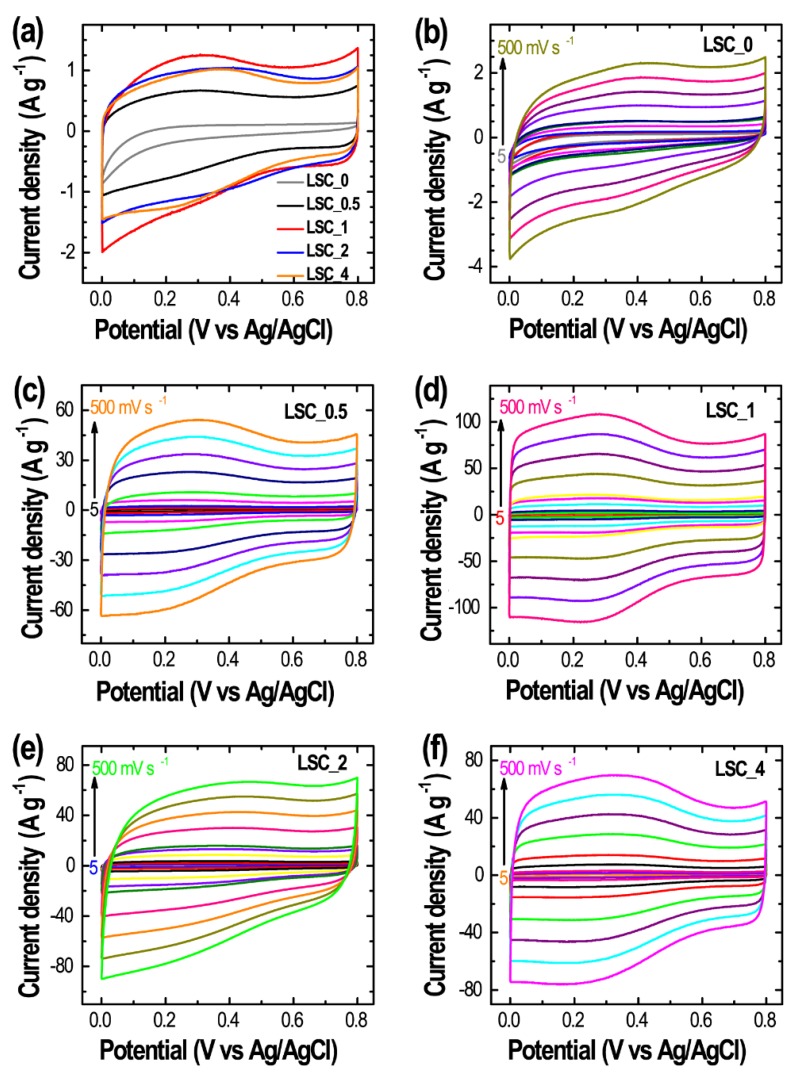
(**a**) Cyclic voltammetry (CV) curves of all the samples at a constant scan rate of 5 mV s^−1^, and the corresponding CV curves at different scan rates from 5 to 500 mV s^−1^. (**b**) **LSC_0**, (**c**) **LSC_0.5**, (**d**) **LSC_1**, (**e**) **LSC_2**, and (**f**) **LSC_4**.

Based on excellent textural parameters and hierarchical micro- and mesoporous architectures as well as the interconnected mesopore structure of the Lapsi seed-derived nanoporous carbon materials, we have investigated their electrochemical supercapacitance performances in the potential range of 0 to 0.8 V in a three-electrode system with an aqueous 1 M H_2_SO_4_ electrolyte. Figure 5a shows the cyclic voltammetry (CV) curves of **LSC_0**, **LSC_0.5**, **LSC_1**, **LSC_2**, and **LSC_4** at a fixed scan rate of 5 mV s^−1^. We have found that the CV responses of all the carbon materials studied here exhibit quasi-rectangular shape, which is typical behavior of EDLCs [61,62]. A slight deviation from rectangular shape is attributed to the presence of oxygen-containing surface functional groups [38]. CV curves indicate that total current collection depends strongly on the ratio of the activating agent. The total current increases with the mixing ratio of Lapsi seed powder and ZnCl_2_ and decreases after attaining a maximum value. That is, the area of the curve initially increases from **LSC_0** to **LSC_1** and then decreases demonstrating the highest energy storage capacity for the **LSC_1** sample, with the area for the CV curves of **LSC_2** and **LSC_4** being comparable but lower (Figure 5a). Figure 5b–f show the current response with **LSC_0**, **LSC_0.5**, **LSC_1**, **LSC_2**, and **LSC_4**_as the electrode materials at different scan rates from 5 to 500 mV s^−1^. The overall current increase with scan rate with the quasi-rectangular shape of CV curve retained indicates the fast electrolyte ion diffusion at the electrode surface which is in turn due to its interconnected mesopore architecture.

**Figure 6:**
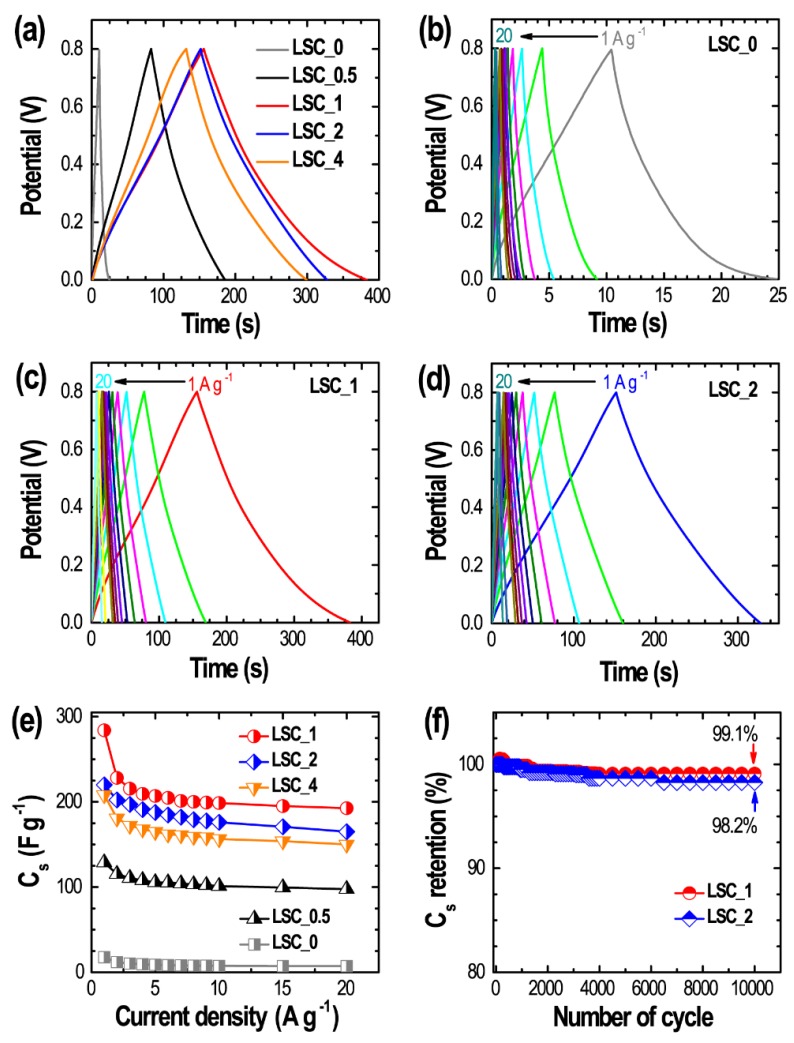
(**a**) Galvanostatic charge–discharge (CD) profiles of all the samples at a constant current density of 1 A g^−1^, (**b**) CD curves of **LSC_0**, (**c**) **LSC_1**, and (**d**) **LSC_2** at different current densities from 1 to 20 A g^−1^ as typical examples. (**e**) Specific capacitance as a function of current density for all the samples, and (**f**) cycling performances of **LSC_1** and **LSC_2** electrodes up to 10,000 charging–discharging cycles.

Further investigation of the electrochemical properties was also carried out by recording galvanostatic charge–discharge (CD) curves at different current densities from 1 to 20 A g^−1^. Figure 6a shows CD curves of **LSC_0**, **LSC_0.5**, **LSC_1**, **LSC_2** and **LSC_4** at a fixed current density of 1 A g^−1^. The typical quasi-triangular shaped CD curves further confirm EDLC behavior of the electrode materials [1,2,3,4]. CD curves further reveal the longest discharge time for **LSC_1** again indicating its improved energy storage capacity over the other samples. Figure 6b–d show the CD curves of **LSC_0**, **LSC_1**, and **LSC_2** at different current densities (1 to 20 A g^−1^) as typical examples. From the CD curves, specific capacitance (*C_S_*) of all the electrode materials was calculated using Equation (1). The *C_S_* were *ca*. 17.8 F g^−1^ (**LSC_0**), 128.8 F g^−1^ (**LSC_0.5**), 284.1 F g^−1^ (**LSC_1**), 220 F g^−1^ (**LSC_2**), and 208 F g^−1^ (**LSC_4**), which is correlated with the surface textural properties. The highest specific capacitance obtained for **LSC_1** can be attributed to the high surface area (2272 m^2^ g^−1^) offered by the hierarchical micro- and mesoporous architecture with interconnected mesopores that allows greater access to a larger quantity of electrolyte ions and also promotes ion diffusion at the electrode surface. The *C_S_* vs. current density curves show high capacitance retention at high current density of 20 A g^−1^ (Figure 6e). **LSC_1** sample retained 67.7% initial capacitance at 20 A g^−1^ demonstrating the high rate capability of the electrode material. We have also tested the cycling stability of selected electrodes (**LSC_1**, and **LSC_2**). Both the electrodes showed excellent cyclic stability with very low capacitance loss even after 10,000 charge–discharge cycles (Figure 6f).

**Figure 7:**
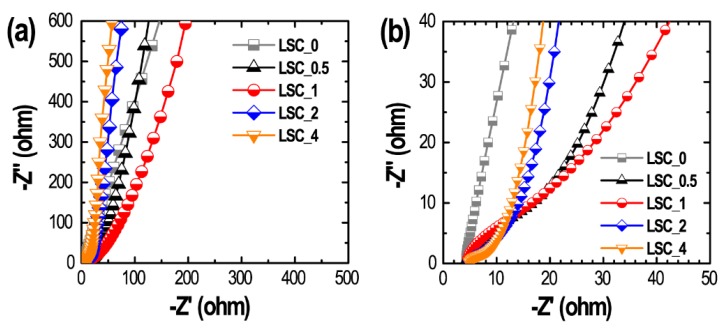
(**a**) Nyquist plots for different electrodes materials (**LSC_0**, **LSC_0.5**, **LSC_1**, **LSC_2**, and **LSC_4**) in 1 M H_2_SO_4_ electrolyte over the frequency range from 0.01 Hz to 100 kHz, and (**b**) corresponding magnified Nyquist plot.

Electrochemical impedance spectroscopy (EIS) was also conducted to investigate the diffusion kinetics of the electrolytes ions, electron-transfer resistance and double-layer charging at the electrode–electrolyte interface. Figure 7 shows Nyquist plots of the ZnCl_2_-activated nanoporous carbon materials. For comparison, EIS profile of the directly carbonized sample **LSC_0** is also included.

The curves show a linear region at low frequency with only weak semicircular responses at high frequency. The weakly semicircular trace in the high-frequency region is an indication of pseudocapacitance originating from the residual oxygen functional groups [63,64]. The first intersection point on the real axis is termed the equivalent series resistance (ESR) which are *ca*. 4.24 Ω (**LSC_0**), 4.43 Ω (**LSC_0.5**), 4.53 Ω (**LSC_1**), 4.87 Ω (**LSC_2**) and 4.77 Ω (**LSC_4**). All the electrode materials exhibit almost similar ESR, which indicates that the difference in the electrochemical performances of these materials is caused by different surface textural properties. 

## 4. Conclusions

In conclusion, we have studied the electrochemical supercapacitance performances of high surface area nanoporous activated carbon materials prepared from Lapsi seed (agricultural waste biomass) through zinc chloride activation at 700 °C. Surface area and pore volume were found respectively in the ranges 931 to 2272 m^2^ g^−1^ and 0.998 to 2.845 cm^3^ g^−1^ depending on the mixing ratio of Lapsi seed powder and ZnCl_2_. Lapsi seed-derived nanoporous carbon materials exhibit excellent electrochemical supercapacitance performance because of their large surface areas, large pore volumes, and interconnected mesopore structures with graphitic carbon structure. The optimal sample exhibited a high specific capacitance of 284 F g^−1^ at 1 A g^−1^ with sustained high rate capability of 67.7% at a high current density of 20 A g^−1^ as well as excellent cycle stability without much capacitance loss even after 10,000 charge–discharge cycles. Our results demonstrate that agricultural waste such as Lapsi seed could be a potential carbon source for the design of nanoporous activated carbon materials having rich surface textural properties making them suitable as electrode materials for high-performance supercapacitor devices [65].

## Figures and Tables

**Table 1:** Porosity properties of the Lapsi seed-derived nanoporous carbon materials.

Sample	*SSA* (m^2^ g^−1^)	*S*_micro_ (m^2^ g^−1^)	*S*_meso_ (m^2^ g^−1^)	*V*_pore_ (cm^3^ g^−1^)	*V*_micro_ (cm^3^ g^−1^)
**LSC_0**	931	412.2	519	1.680	0.868
**LSC_0.5**	1482.5	1289.9	193	0.998	0.710
**LSC_1**	2272.3	1948.2	324	1.611	1.159
**LSC_2**	1524.5	975.1	549	1.622	0.939
**LSC_4**	1696.2	986.9	710	2.845	1.50

*SSA* = specific surface area, *S*_micro_ = micropore surface area, *S*_meso_ = mesopore surface area, *V*_pore_ = total pore volume, and *V*_micro_ = micropore volume obtained from the DFT method.
